# Reasons for Failed Mechanical Thrombectomy in Posterior Circulation Ischemic Stroke Patients

**DOI:** 10.1007/s00062-020-00950-x

**Published:** 2020-09-07

**Authors:** Charlotte S. Weyland, Ulf Neuberger, Arne Potreck, Johannes A. R. Pfaff, Simon Nagel, Silvia Schönenberger, Martin Bendszus, Markus A. Möhlenbruch

**Affiliations:** 1 Department of Neuroradiology, Heidelberg University Hospital, Im Neuenheimer Feld 400, 69120 Heidelberg, Germany; 2 Department of Neurology, Heidelberg University Hospital, Im Neuenheimer Feld 400, 69120 Heidelberg, Germany

**Keywords:** Rescue strategies, Alternative vascular access, Local thrombolysis, Intracranial stenting, Mortality

## Abstract

**Background and Purpose:**

To determine reasons for failed recanalization in mechanical thrombectomy (MT) of the posterior circulation.

**Methods:**

Retrospective single center analysis of reasons for MT failure in the posterior circulation. Failed MTs were categorized according to the reason for procedure failure in failed vascular access, failed passage of the target vessel occlusion and MT failure after passing the occluded target vessel. Patient characteristics were compared between failed and successful MT.

**Results:**

Patients with failed MT (30/218 patients, 13.8%) were categorized into futile vascular access (13/30, 43.3%), abortive passage of the target vessel occlusion (6/30, 20.0%) and MT failure after passing the vessel occlusion (11/30, 36.7%). In 188/218 (86.2%) successful MTs alternative vascular access, local intra-arterial (i.a.) thrombolysis and emergency stent-assisted PTA prevented 65 MT failures. Patients with failed MT showed a higher NIHSS at discharge, a higher pc-ASPECTS in follow-up imaging, a higher mRS 90 days after stroke onset and a high mortality rate of 77.0% (mRS at 90 days, median (IQR): 6 (6–6) vs. 4 (2–6) for successful MT, *p*-value < 0.001). Co-morbidities and stroke etiology were not different compared to sufficient recanalization with atherosclerotic disease as the leading stroke etiology in both groups.

**Conclusion:**

Failure of MT in posterior circulation ischemic stroke patients is associated with a high mortality rate. Reasons for MT failure are diverse with futile vascular access and MT failure after passing the vessel occlusion as the leading causes. Alternative vascular access, local i.a. thrombolysis and stent-assisted PTA can prevent MT failure.

## Key Points

- Mechanical thrombectomy (MT) of the posterior circulation is technically successful in over 85% of this population and associated with a very high mortality rate in cases of MT failure.
- Reasons for MT failure in the posterior circulation are diverse with futile vascular access and failed MT after passage of the target vessel occlusion as leading causes before abortive passage of the target vessel occlusion.
- Alternative vascular access, local i.a. thrombolysis and stent-assisted PTA prevent MT failure in selected patients.

## Introduction

Mechanical thrombectomy (MT) has become the state of the art for treating patients with ischemic stroke and large vessel occlusion (LVO) in the anterior circulation since several randomized, multicenter studies emerged in 2015 proved its superiority to intravenous rt-PA alone [1–4]. A successful recanalization of the target vessel occlusion is crucial for a good clinical outcome and with modern thrombectomy techniques, it lies over 80% [5]. Recently, models emerged to classify the considerable minority of failed recanalization for further development of rescue strategies and to increase the MT success rate in the anterior circulation [6, 7]. Failed recanalization attempts are subdivided into interventions with futile vascular access and failed thrombus removal. The second group is further classified as failure to pass the target vessel occlusion and as stent-retriever failure, where the thrombus cannot be removed despite all MT maneuvers. Studies following this approach investigated causes for recanalization failure in MT of the anterior circulation [8] and for stent-retriever-based thrombectomy also focusing on the anterior circulation [9]. Rescue strategies, such as intracranial stenting and alternative vascular access, are discussed for failed MT of the anterior circulation.

The use of MT for large vessel occlusions of the posterior circulation does not currently have the same level of evidence. Evidence for its safety and efficacy using modern thrombectomy techniques can be derived from smaller studies as well as evidence for a very high mortality rate when MT of the posterior circulation fails [10, 11]. Technically successful recanalization of patients with posterior circulation stroke and MT represents an independent predictor for good functional outcome [12, 13]. Nevertheless, poor outcome despite successful recanalization, so called futile recanalization, occurs more often compared to MT of the anterior circulation [12, 14–16]. The rate of sufficient recanalization is reported to be around 80% and comparable to LVO of the anterior circulation for adjusted patient cohorts [16, 17]. The aim of this study was to investigate outcome and reasons for MT failure in the posterior circulation and determine possible predictors of procedure failure.

## Methods

The prospective stroke database of Heidelberg’s comprehensive stroke center was searched for all patients with acute ischemic stroke of the posterior circulation and intention to perform MT between March 2009 and April 2019. All cases included were conducted with modern MT techniques at hand starting with the Solitaire AB stent (Medtronic, Dublin, Ireland) in 2009 and continuously modified by adding or exchanging with new material.

Due to the retrospective character of this study written informed consent was waived. There was no age restriction or other patient selection criteria prior to study analysis. All imaging parameters (e.g. pc-ASPECTS, localization of target vessel occlusion, reperfusion result) were obtained defined by the neuroradiologists (neuroradiologist in training and specialist with at least 6 years radiologic experience) of the in-house stroke team and revised for this study by neuroradiologists with 5 and 3 years of training. All clinical parameters were obtained by the neurologists of our in-house stroke team and after discharge (mainly for mRS 90 days) with telephone interviews conducted by mRS/NIHSS certified medical doctors.

### Decision Making and Standard Endovascular Procedure for MT of the Posterior Circulation

The consensual decision for MT of the posterior circulation was made by the neurointerventionalist and an experienced neurologist. The decision for MT was based on current stroke guidelines. The MT was performed by an experienced neuroradiologist (at least 5 years of training in diagnostic and interventional radiology) with the assistance of a second neuroradiologist in training. The standard interventional approach is a femoral access (7F Flexor Shuttle Guiding Sheath 80 cm, Cook, Bloomington, IN, USA), a 5–6F intermediate catheter for access to the dominating or occluded vertebral artery and a microcatheter in a triaxial system for stent-retriever thrombectomy under continuous aspiration or direct aspiration.

The choice of catheters and MT maneuver approach (direct thrombus aspiration as first attempt or stent-retriever thrombectomy in solumbra technique) was made by the interventionalist. The thrombectomy technique is changed when a thrombectomy strategy fails (e.g. from aspiration to stent-retriever thrombectomy). As standard approach up to three different stent-retriever devices are used in our facility with up to three attempts per device before terminating unsuccessful MT. The material mostly used in this cohort is a 5F-Neuron intermediate catheter (2009–2013; Penumbra, Alameda, CA, USA) or 5F/6F-Sofia intermediate catheter (2013 until today; Microvention, Aliso Viejo, CA, USA). All modern stent-retriever models are available in our facility with Solitaire (Medtronic, Boulogne Billancourt, France), Trevo (Stryker, Kalamazoo, MI, USA) and pREset (Phenox, Bochum, Germany) used most commonly for the posterior circulation. The MT of the posterior circulation is performed with the patient under intubation anesthesia. For rescue stenting during MT of persistent basilar artery occlusions a glycoprotein (GP) IIb/IIIa inhibitor is given for 6 h during and after intervention followed by an overlapping loading with double platelet inhibition with aspirin and clopidogrel.

### Classification for Reasons of Failed MT

The following classification systems for failed recanalization in MT of the anterior circulation, all cases of failed MT were categorized in one of the following groups.

#### Category I: Target Vessel Occlusion not Reached

##### Ia

The intracranial target vessel could not be reached due to difficult aortic anatomy, vessel tortuosity and/or vertebral hypoplasia.

##### Ib

Target occlusion was not reached due to atherosclerotic stenosis/occlusion of the vertebral arteries.

#### Category II: Target Vessel Occlusion Reached but not Passed

##### II

The intracranial target vessel was reached but could not be passed for any MT maneuver until the end of the procedure.

#### Category III: Target Vessel Occlusion Reached and Passed

##### IIIa

Target occlusion stayed occluded despite MT maneuvers or vessel lumen reoccluded until the end of the MT.

##### IIIb

After passing the vessel occlusion complications or neurologic deterioration of the patient lead to a termination of the procedure.

### pc-ASPECTS and Reperfusion Result—For MT of the Posterior Circulation

The Alberta Stroke Program Early CT Score (ASPECTS) was originally developed as a reliable topographic assessment for early ischemic signs of infarct tissue in the middle cerebral artery territory with predictive value [18]. Later the score was modified for the posterior circulation, the so called pc-ASPECTS [19], which is a powerful predictor for functional outcome when transferred to diffusion-weighted MRI for ischemic lesions in the posterior circulation [20]. We determined the pc-ASPECTS in admission and follow-up imaging. Due to changing imaging protocols during the assessment period of this study and varying clinical settings CT and MR imaging were both evaluated depending on availability.

The reperfusion result after MT was reviewed independently case by case. A persistent occlusion of the target vessel was considered as failed MT (comparable to modified thrombolysis in cerebral infarction score 0–1 in the anterior circulation).

### Statistical Analysis

Descriptive group statistics were gathered for patients with either successful or failed MT. Quantitative data were tested for normal distribution using the Shapiro-Wilk normality test and showed that all quantitative data were distributed non-parametrically. Univariate analyses were performed using Wilcoxon rank-sum and χ^2^-tests for nonparametric quantitative variables and categorical variables, as appropriate, to compare all parameters between groups of successful and failed MT. A *p*-value of less than or equal to 0.05 was considered to indicate a significant difference. All statistical analyses were performed by using R version 3.6.0 (Foundation for Statistical Computing, Vienna, Austria).

## Results

Overall, 1671 patients underwent MT for acute ischemic stroke in our department between March 2009 and April 2019. Of the patients 218 were treated for ischemic stroke of the posterior circulation. In 30/218 patients (13.8%) the MT failed and 188/218 patients (86.2%) showed a sufficient recanalization result after MT. The locations of the target vessel occlusion, which involved the basilar artery in all cases (intracranial vertebral artery to proximal basilar artery, midbasilar artery occlusion or distal basilar artery to posterior cerebral artery occlusion) were represented equally in the groups of sufficient and failed MT (Table 1). There was no tendency towards a higher success rate in more recent years while the overall number of MTs performed gradually increased.

**Table 1:** Mechanical thrombectomy in the posterior circulation grouped according to reperfusion result

	Failed MT (*n* = 30)	Successful MT (*n* = 188)	*p*-value
*Age (years), mean (SD)*	71 (13)	73 (14)	0.35513
*Male, n (%)*	19 (63.3)	110 (58.5)	0.76488
*Coronary artery disease, n (%)*	9 (34.6)	49 (27)	0.55894
*Known atrial fibrillation*	13 (50)	71 (39)	0.39278
*Arterial hypertension, n (%)*	25 (93)	140 (77)	0.10723
*Diabetes mellitus II, n (%)*	6 (23)	40 (21.9)	1
*Hypercholesterinemia, n (%)*	12 (46)	62 (34)	0.32446
*Stroke-related clinical and imaging aspects*			
*pmRS, median (IQR)*	1 (0–2)	1 (0–2)	0.72645
*mRS 90* *days after stroke onset, median (IQR)*	6 (6–6)	4 (2–6)	*0.00029*
*Mortality, n (%)*	23 (77.0)	68 (36.2)	*0.001*
*Initial NIHSS score, median (IQR)*	14 (8–27)	22 (10–35)	0.13093
*NIHSS at discharge, median (IQR)*	34 (11–34)	8 (3–34)	*0.00005*
*Pc-ASPECTS baseline, median (IQR)*	8 (7–10)	9 (7–10)	0.63243
*Pc-ASPECTS follow-up, median (IQR)*	5 (3–7.5)	7 (5–8)	*0.03049*
*Intravenous rtPA, n (%)*	13 (43.3)	104 (56)	0.27753
*Time of symptom onset known, n (%)*	19 (63.3)	131 (69.6)	0.62788
*Etiology of stroke*			
*Large artery atherosclerosis, n (%)*	14 (46.7)	62 (33)	0.17548
*Cardioembolism, n (%)*	10 (33.3)	76 (41)	0.57639
*Small vessel occlusion, n (%)*	0	0	–
*Other determined etiology, n (%)*	2 (6.7)	17 (9)	0.92973
*Embolic stroke of unknown source, n (%)*	4 (13.3)	23 (12.3)	1
*Unknown etiology, n (%)*	0	15 (8)	0.2224
*Procedural aspects*			
*Time from stroke onset to groin puncture, in minutes, median (IQR)*	388 (229–713)	329 (200–615)	0.33534
*Procedure time (groin puncture to reperfusion result) in minutes, median (IQR)*	139 (50–189)	73 (42–111)	0.10029
*Location of occlusion*			
*V4-segment of VA to proximal BA, n (%)*	5 (17)	19 (10)	0.45205
*Mid-BA, n (%)*	24 (80)	151 (80)	1
*Distal BA to PCA, n (%)*	5 (9.5)	34 (18)	1
*Number of direct aspirations, median (IQR)*	0	0	–
*Number of stent-retriever thrombectomy under continuous aspiration, mean (SD)*	0 (0–3)	1 (1–3)	*0.03579*
*Stent-assisted PTA of VA/BA, n (%)*	9 (30)	56 (30)	1

*BA* basilar artery, *IQR* interquartile range, *mRS* modified Rankin Scale, *NIHSS* National Institute of Health Stroke Scale, *PCA* posterior cerebral artery, *pc-ASCPETS* posterior-circulation Alberta Stroke Program Early CT Score, *PTA* percutaneous transluminal angioplasty, *rtPA* recombinant tissue Plasminogen Activator, *SD* standard deviation, *VA* vertebral artery

### Patient Characteristics Primary Analysis

Baseline characteristics and demographics in comparison to failed MT and successful MT are shown in Table 1. There was no significant difference for the stroke-related comorbidities coronary artery disease, diabetes, known atrial fibrillation, arterial hypertension or hypercholesterinemia. The most frequently determined stroke etiology according to the Trial of Org 10172 in Acute Stroke Treatment (TOAST) classification were large artery atherosclerosis (TOAST 1, failed MT: 46.7%, successful MT: 33.0%) and cardioembolism (TOAST 2, failed MT: 33.3%, successful MT: 41.0%) with no statistical difference between the two groups (*p*-value: 0.175 and 0.576, respectively). Stent-assisted PTA was performed in 30.0% of successful (56 patients) and 30.0% of failed (9 patients) MTs, the patients neurological deficit (per NIHSS) and handicap (per mRS) were different with a higher NIHSS at discharge for patients with failed MT (median (IQR) failed MT 34 (11–34); successful MT 2/3 8 (3–34), *p*-value <0.001), a higher mortality rate after failed MT (*n* = 23/30, 77%) and a higher mRS 90 days after stroke onset (median (IQR) failed MT 6 (6–6), successful MT 4 (2–6), *p*-value <0.001). Also, the pc-ASPECTS in the follow-up imaging (CT or MRI, 1–3 days after MT) was significantly lower after failed MT (median IQR failed MT 5, 3–7.5, successful MT 7, 5–8). In both groups, a stent-assisted percutaneous transluminal angioplasty (PTA) was performed in 30.0% of the cases during the MT.

### Failed Mechanical Thrombectomy

#### Category I

In 12/30 cases (40.0%) of failed MT, an unsuccessful vascular access was the reason for procedure failure. Atherosclerotic stenosis or occlusion of the vertebral arteries was the cause in seven cases (category Ia). Anatomically difficult access with aortic or vertebral artery elongations was the cause in five cases (category Ib). In overall 7/218 MTs of the posterior circulation an alternative vascular access was performed after the standard femoral access failed. Four times a brachial/radial access followed after a failed femoral access. This led to three successful MTs and one failed MT. A retrograde access through a posterior communicating artery was performed two times for local, intra-arterial rtPA application ensuing one successful MT. One time the vertebral artery was directly accessed in the V2 segment through a dominant ascending cervical artery and a successful MT followed.

#### Category II

In 7/30 cases (23.3%) the target vessel occlusion could not be passed with a probable underlying atherosclerotic occlusion stated by the interventionalist in five of these cases. Local intra-arterial (i.a.) thrombolysis (rtPA) was performed in 13/218 cases. Four times a persisting basilar artery occlusion was treated with i.a. rtPA with two successful recanalization results. Six times i.a. rtPA and an additional stent-assisted PTA was performed. Three times an embolic vessel occlusion (PCA and SUCA) that occurred after recanalization of the basilar artery were targeted.

#### Category III

In 11/30 cases (36.7%), the MT failed after passing the occluded vessel. In three of these cases, the vessel stayed occluded or the vessel lumen reoccluded despite MT maneuvers (category IIIa). An emergency stent-assisted PTA was conducted if possible in these scenarios. Stenting of the V4 segment of the vertebral artery and/or the basilar artery was performed in 65/218 cases of MT in the posterior circulation. In 9/65 cases MT failed comprising three device malfunctions (i.e. stent kinking) and six cases where the stent implantation did not lead to a restoration of the target vessel volume.

In five cases, intraprocedural complications (two cases of vessel perforation and three cases of stent kinking or failed stent unfolding) or the patient’s deteriorating clinical status during the MT let to a termination of the intervention (category III B).

## Discussion

In this single center analysis we showed that futile vascular access due to atherosclerotic disease or anatomic difficulties (*n* = 12/30 patients, 40.0%) and MT failure after passing the target vessel occlusion (*n* = 11/30 patients, 37.0%) are the leading causes for failed recanalization in posterior circulation MT, followed by abortive passage of the thrombus (*n* = 7/30 patients, 23.0%).

Patients with MT failure showed a higher mRS 90 days after stroke onset with a high mortality rate of 77.0% and a very high NIHSS at discharge as well as a lower pc-ASPECTS in follow-up imaging. No specific target vessel occlusion side, which involved the basilar artery in all cases in this cohort, was associated with a higher risk of MT failure. Corresponding to studies of the anterior circulation, further investigation is needed to identify probable associations of thrombus length or texture and MT failure [21, 22]. Overall, a sufficient recanalization result for stroke patients and MT of the posterior circulation was achieved in 86.2%, which is comparable to the 90.3% and 84.5% success rate of MT in basilar artery occlusions published by Meinel et al. and Sun et al., respectively, and underlines the notion that success rates for MT in the posterior circulation are comparable to the anterior circulation [16, 23].

Likewise, reasons for MT failure are diverse compared to studies on MT failure in anterior circulation ischemic stroke patients with a similar tendency towards a futile vascular access and failed MT after passing the target vessel occlusion as the leading categories. Futile vascular access is a common reason for MT failure in the anterior and posterior circulations, which highlights the necessity to discuss alternative vascular access routes in MT as a rescue strategy. In individual cases of this cohort an alternative access led to a successful target vessel recanalization. These alternative accesses comprised brachial/radial accesses, retrograde access through a dominant posterior communicating artery and access from a dominant ascending cervical artery to the vertebral artery’s V3/4 segment. An unsuccessful passage of the target vessel occlusion as reason for a failed MT could only be witnessed in a few cases and was seldom successfully treated with a local intra-arterial lysis (rtPA) [8, 9]. Also, MT failure after passing the target vessel occlusion seems to be equally relevant in anterior and posterior circulation MT; however, this scenario is linked to different reasons in the posterior circulation. In the anterior circulation remaining target vessel occlusion after trying different stent-retriever devices, the so-called true stent-retriever failure, is the main reasons for MT failure after passing the target vessel occlusion. Stent-assisted PTA during MT is discussed as possible rescue strategy but remains an exception to the rule so far [8].

In the posterior circulation, however, emergency stent-assisted PTA is already performed in a great number of MTs (65/218 MTs in this cohort) preventing many cases of MT failure but also failing itself in 14% (9/65 cases). The implementation of intracranial stent-assisted PTA during MT of the posterior circulation therefore often seems inevitable but also represents an existing source of peril. Therefore, to improve the success rate and safety of MT in the posterior circulation overall, devices for stent-assisted PTA need further improvement. Although patient comorbidities and stroke etiology were not different between the two groups of successful and failed MT in the posterior circulation, the high rate of large vessel atherosclerosis as stroke etiology in both groups (33.0% and 46.7% for successful and failed MT, respectively) reflects a high significance of atherosclerosis for posterior circulation stroke patients. Arguably, detecting and treating atherosclerotic stenosis of the basilar artery before ischemic stroke for patients at risk can be seen as primary prevention. Primary stenting for high-grade basilar artery stenosis is proven to be successfully performed in cases of refractory medical treatment [24, 25]. Risk stratification for these patients is currently not available. Strengthening strategies to prevent MT failure of the posterior circulation represents in many cases the only option to decrease the high mortality rate of failed MT of basilar artery occlusions. The majority of patients with failed MT in this cohort were comatose on discharge to another hospital or medical facililty, or had died before discharge. Thus, palliative or rehabilitative concepts can seemingly only contribute to a better outcome in selected patients.

### Limitations

This single center retrospective study with a substantial patient cohort (30 failed MT in 218 patients for MT of the posterior circulation) can serve as a first approach for investigating reasons of failed MT in the posterior circulation. Future prospective studies should focus on investigating important issues that remain unclear, such as the management and implementation of stent-assisted PTA in failed MT of the posterior circulation, to further improve therapeutic options in these patients. Although this study cohort did not show a decrease of MT failure or complications over the years in the wake of increasing MT cases overall, the growing experience with MT for anterior and posterior circulation target vessel occlusions as standard intervention in modern endovascular stroke treatment can influence the findings.

## Conclusion

Mechanical thrombectomy of the posterior circulation is successful in the majority of patients but shows a high mortality rate if recanalization fails. Reasons for MT failure are diverse, with futile vascular access and MT failure after passing the target vessel occlusion as leading causes. Alternative vascular access, local i.a. thrombolysis and stent-assisted PTA can prevent MT failure. Further investigation and device innovation are necessary to improve the success rate of MT particularly regarding the frequently performed emergency stent-assisted PTA.

## Abbreviations

BA: Basilar artery
LVO: Large vessel occlusion
MT: Mechanical thrombectomy
mRS: Modified Rankin scale
NIHSS: National Institutes of Health Stroke Scale
PCA: Posterior cerebral artery
pc-ASPECTS: Posterior circulation Alberta Stroke Early CT Score
PTA: Percutaneous transluminal angioplasty
rtPA: Recombined tissue plasminogen activator
TOAST: Trial of Org 10172 in Acute Stroke Treatment
VA: Vertebral artery
